# Hydrophobicity Influence on Swimming Performance of Magnetically Driven Miniature Helical Swimmers

**DOI:** 10.3390/mi10030175

**Published:** 2019-03-06

**Authors:** Chengwei Ye, Jia Liu, Xinyu Wu, Ben Wang, Li Zhang, Yuanyi Zheng, Tiantian Xu

**Affiliations:** 1 School of Science and Engineering, The Chinese University of Hong Kong, Shenzhen 518172, China; chengweiye@link.cuhk.edu.cn; 2 Guangdong Provincial Key Laboratory of Robotics and Intelligent System, Shenzhen Institutes of Advanced Technology, Chinese Academy of Sciences, Shenzhen 518055, China; jia.liu1@siat.ac.cn (J.L.); xy.wu@siat.ac.cn (X.W.); 3 CAS Key Laboratory of Human-Machine Intelligence-Synergy Systems, Shenzhen Institutes of Advanced Technology, Shenzhen 518055, China; 4 Department of Mechanical and Automation Engineering, The Chinese University of Hong Kong, Hong Kong, China; wangbben@gmail.com (B.W.); lizhang@cuhk.edu.hk (L.Z.); 5 Shanghai Jiaotong University, Shanghai 200233, China; zhengyuanyi@163.com; 6 Shenzhen Key Laboratory of Minimally Invasive Surgical Robotics and System, Shenzhen Institutes of Advanced Technology, Chinese Academy of Sciences, Shenzhen 518055, China

**Keywords:** magnetically driven helical swimmer, step-out frequency, low Reynolds number, hydrophilibity influence

## Abstract

Helical microswimmers have been involved in a wide variety of applications, ranging from in vivo tasks such as targeted drug delivery to in vitro tasks such as transporting micro objects. Over the past decades, a number of studies have been established on the swimming performance of helical microswimmers and geometrical factors influencing their swimming performance. However, limited studies have focused on the influence of the hydrophobicity of swimmers’ surface on their swimming performance. In this paper, we first demonstrated through theoretical analysis that the hydrophobicity of swimmer’s surface material of the swimmer does affect its swimming performance: the swimmer with more hydrophobic surface is exerted less friction drag torque, and should therefore exhibit a higher step-out frequency, indicating that the swimmer with more hydrophobic surface should have better swimming performance. Then a series of experiments were conducted to verify the theoretical analysis. As a result, the main contribution of this paper is to demonstrate that one potential approach to improve the helical microswimmers’ swimming performance could be making its surface more hydrophobic.

## 1. Introduction

Due to the recent advances in microfabrication brought by the rise of new manufacturing methods [1,2,3,4,5], as well as the profound innovation in motion control systems [6,7,8,9,10], the field of helical microswimmers has been undergoing rapid developments. Helical microswimmers are playing an increasingly essential role in a wide variety of applications, ranging from in vivo tasks such as minimally invasive surgery and targeted gene delivery [11,12,13,14,15], to in vitro tasks such as photocatalytic water purification and transporting micro objects [16,17]. Among several approaches to actuate and manipulate the microswimmers, the use of magnetic fields emerges to be the most auspicious option, since it supports various swimming mechanisms and prevents microswimmers from adversely interacting with tissues [18].

Purcell studied on the swimming techniques of natural swimmers in his 1977 work [19], where he presented in detail one common solution to the problem of swimming at low Reynolds number: the corkscrew-like rotating propulsion approach adopted by *E. coli* bacteria. The rotary motor of *E. coli* bacteria drives its bundle of flagella to rotate in a helical pattern, and in this way generates propulsion. Nowadays helical microswimmers adopt the same swimming mechanism as *E. coli* bacteria, substituting the internal rotary engine with external magnetic field.

In the past decades, researchers have designed and fabricated various types of helical microswimmers with different head shapes, magnetic positioning, as well as geometrical parameters of head and tail. In addition, a number of studies have been established on the influence of those variables on the swimming performance of helical microswimmers. In 2015, Xu et al. proposed that the magnetic positioning of helical microswimmers affects the number of possible magnetization directions and further results in different rotational propulsion characteristics (gentle cut-off, brutal cut-off, or frequency saturation), and that the head shape only affects the values of cut-off frequency with no influence on the rotational propulsion characteristics [20]. What is more, it is demonstrated by experiments that the pitch and the number of turns positively contribute to the swimming performance of helical microswimmers, while the width and the thickness negatively influence the swimming performance, and that the pitch is the most significant factor among the four geometric parameters [21]. However, limited attention has been focused on the role of surface materials in affecting the swimming performance. In parallel with the contribution of coefficient of friction (COF) to resistive force, it is reasonable to expect that discrepancies in surface material characteristics, such as hydrophobicity or roughness, would to some extent result in different swimming performance.

This paper aims to study the influence of surface material hydrophobicity of miniature helical swimmers on their swimming performance at low Reynolds number, especially their step-out frequency values. We first presented a theoretical analysis on how the hydrophobicity of surface material would affect its swimming performance, and then verified it through a series of experiments. Experiments are conducted in glycerine solutions of different fluid viscosity, using two helical swimmers which are identical except for their surface material hydrophobicity.

In the following parts of the paper, Section 2 introduces the background theories regarding the dynamics of helical microswimmers, and theoretically predicts how the surface material hydrophobicity would affect the swimming performance of helical microswimmers. Section 3 presents the system overview including the helical swimmers with different hydrophobicity surface and the magnetic actuation system employed in experiments. Section 4 explains the design of experiments, displays the results, and presents analysis regarding the impact of hydrophobicity and fluid viscosity on step-out frequency. Section 5 overviews the work accomplished in this paper and provides future prospects.

## 2. Theory

### 2.1. Magnetic Actuation

In the experiments, a uniform rotating magnetic field is applied to drive and control the helical microswimmer. Within such a magnetic field, the magnetic torque and the magnetic force exerted on the swimmer are given by [22]:

(1) $$T_{B}=\vartheta M\times B$$

(2) $$F_{B}=\vartheta \left(M\;\cdot \;\nabla \right)B$$

where ϑ denotes the volume of the helical microswimmer, *M* denotes the average magnetization of the helical microswimmer, and *B* denotes the flux density of the applied magnetic field. Note that both equations rely on the assumption that the spatial changes in magnetic field must be small enough so that it can be regarded as uniform throughout the volume of the helical microswimmer.

Since the applied magnetic field generated by the 3D Helmholtz coil system is uniform, the magnetic force $F_{B}$ is constantly zero, therefore the microswimmer is actuated and controlled by the magnetic torque with no magnetic force.

The magnetic torque $T_{B}$ serves as the driving torque for swimmer locomotion; it tends to align the magnetization of the helical microswimmer with the direction of the applied field and drive it to rotate around its axis synchronously with the rotating magnetic field. To guarantee steady-state locomotion of the swimmer, the applied magnetic torque has to counterbalance the resistive torques exerted on the swimmer due to fluidic drag. The‘ total fluidic drag experienced by the swimmer is composed of two parts: the form drag and the skin friction drag [23], which will be discussed in the following two sections.

### 2.2. Form Drag: Resistive Force Theory

**Figure 1:**
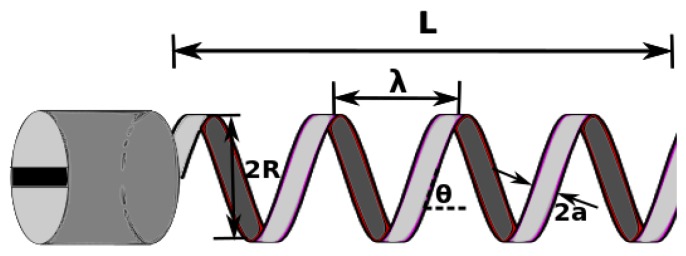
Helical microswimmer model.

When modeling the propulsion of a spermatozoon, Gray and Hancock [24] considered the forces exerted on an infinitely short element δs by virtue of its transverse velocity *V*. The drag force δF is composed of two parts: a tangential drag force $\delta F_{∥}$ and a normal drag force $\delta F_{⊥}$, which can be respectively expressed as:

(3) $$\delta F_{∥}=C_{T}V\sin\varphi \delta s$$

(4) $$\delta F_{⊥}=C_{N}V\cos\varphi \delta s$$

where φ is the orientation of the infinitely short element with respect to the axis of propulsion, while $C_{T}$ and $C_{N}$ are the drag coefficients in tangential direction and in normal direction, respectively. They can be expressed as:

(5) $$C_{T}=\frac{2\pi \mu }{\ln\frac{2\lambda }{a}-\frac{1}{2}}$$

(6) $$C_{N}=\frac{4\pi \mu }{\ln\frac{2\lambda }{a}+\frac{1}{2}}$$

with μ being the fluid viscosity, λ being the pitch of the helical tail, and *a* being the filament radius of the helical tail. For a microswimmer with a helical tail of length *L*, radius *R*, and pitch angle θ, as is shown in Figure 1, the total fluidic drag force *D* and the total fluidic drag torque *T* exerted on it are derived by integrating the forces and torques on infinitely small elements along the length of its helical tail. They are given by [25]:

(7) $$D=U\left(C_{N}\sin^{2}\theta +C_{T}\cos^{2}\theta \right)\frac{L}{\cos\theta }$$

(8) $$T=\left(\Omega R^{2}\right)\left(C_{N}\cos^{2}\theta +C_{T}\sin^{2}\theta \right)\frac{L}{\cos\theta }$$

where *U* and Ω describe the swimming behavior of the helical microswimmer, with *U* denoting the axial velocity and Ω denoting the rotation rate.

### 2.3. Skin Friction Drag

For a body swimming in viscous fluid, the skin friction drag force exerted on an infinitely small area dA of it is given by [26]:

(9) $$dF=C_{f}\frac{\rho v^{2}}{2}dA$$

where ρ denotes the fluid density, *v* denotes the fluid speed relative to body surface, and $C_{f}$ denotes the skin friction drag coefficient, which can be expressed as:

(10) $$C_{f}=\frac{2\tau_{w}}{\rho v^{2}}$$

with $\tau_{w}$ being the wall shear stress. Combining the previous two equations, the skin friction drag force exerted on the whole body can be obtained by integrating the wall shear stress over the body surface. The skin friction drag force applies in tangential direction, and its corresponding moment arm with respect to body center line is equal to the radius of helical tail, denoted by *R*. Thus the total skin friction drag force and drag torque are given by:

(11) $$F=\int_{S}dF=\int_{S}\tau_{w}dA$$

(12) $$T=RF=R\int_{S}\tau_{w}dA$$

According to Newton’s law of viscosity [27], the shear stress between adjacent fluid layers is proportional to the velocity gradient between the two layers, with the proportionality constant being fluid viscosity. The wall shear stress $\tau_{w}$ is proportional to the velocity gradient at the body surface. A more hydrophobic surface has a larger contact angle, which results in less solid-water surface contact. Since the increased solid-water surface contact contributes to a larger velocity gradient [28], the velocity gradient at the more hydrophobic surface is relatively smaller, and thus the shear stress is lower.

### 2.4. Dynamics of Helical Microswimmers and Step-Out Frequency

As is discussed in previous sections, a pair of opposing torques are controlling the swimming behavior of the helical microswimmer. The driving torque is the magnetic torque, which tends to drive the swimmer to rotate in sync with the rotating magnetic field; while the resistive torque is the fluidic drag torque, which consists of form drag torque and skin friction drag torque.

While swimming in viscous fluid, a helical microswimmer exhibits a step-out frequency, which marks a crucial turning point of its swimming behavior [20]. When the rotation frequency of the magnetic field is below the step-out frequency, the driving magnetic torque is large enough to counterbalance the resistive fluidic drag torque, which enables the swimmer to rotate synchronously with the magnetic field, and its swimming velocity increases in an approximately linear pattern with respect to the increasing rotation frequency. When the rotation frequency of the magnetic field is beyond the step-out frequency, the magnetic torque is insufficient to offset the drag torque, therefore the swimmer rotates asynchronously with the applied field, and its swimming velocity undergoes a drastic and non-linear decline as the rotation frequency goes up [22]. Thus the step-out frequency is essentially the largest frequency value at which the balance between magnetic torque and fluidic drag torque still holds. Given Equations (1), (8), and (12), the torque balance at this point can be written as:

(13) $$\vartheta M\times B=\left(2\pi f_{0}R^{2}\right)\left(C_{N}\cos^{2}\theta +C_{T}\sin^{2}\theta \right)\frac{L}{\cos\theta }+R\int_{S}\tau_{w}dA$$

where $f_{0}$ denotes the step-out frequency. With simple transformation:

(14) $$f_{0}=\frac{\vartheta M\times B-R\int_{S}\tau_{w}dA}{\left(2\pi R^{2}\right)\left(C_{N}\cos^{2}\theta +C_{T}\sin^{2}\theta \right)\frac{L}{\cos\theta }}$$

As is discussed in Section 2.3, for the more hydrophobic surface, the less solid-water surface contact results in a smaller velocity gradient, and thus gives a lower shear stress $\tau_{w}$. Note that the above equation indicates that the shear stress $\tau_{w}$ negatively influences the step-out frequency $f_{0}$, so a lower shear stress is supposed to result in a larger step-out frequency value. Therefore, we are expecting to observe a relatively smaller $f_{0}$ value for the less hydrophobic microswimmer and a relatively larger $f_{0}$ value for the more hydrophobic microswimmer in the following experiments.

## 3. System Overview

### 3.1. Magnetic Actuation System

**Figure 2:**
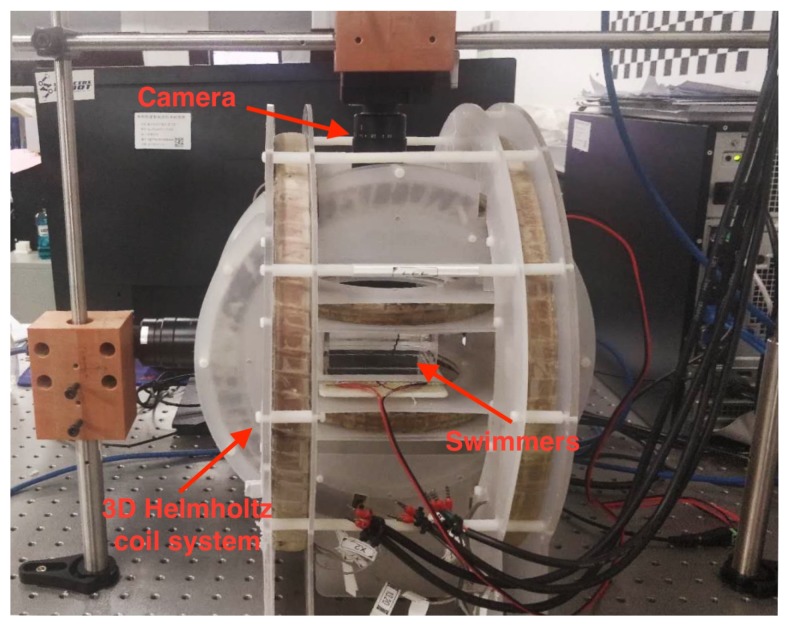
Experiment platform.

In the experiments, the uniform rotating magnetic field used to actuate and control the helical microswimmers is generated by a 3D Helmholtz coil system shown in Figure 2, which is composed of three orthogonally arranged Helmholtz coil pairs. Each Helmholtz coil pair is driven by a Maxon ESCON 70/10 motor driver (Maxon Motor, Sachseln, Switzerland). To control the swimming behaviors (e.g., advancing velocity and heading direction) of the microswimmer, a PC program sends out current signals, which pass through a Sensoray S826 PCIe A/D IO card (Sensoray, Tigard, OR, USA) and reach the motor drivers, so that the analog communication between PC and the motor drivers is realized. The coil system is capable of generating a uniform field with magnetic flux density of 6.85 mT at 2 A, throughout the working space of size 80 mm × 50 mm × 40 mm.

An associative camera (PointGreyGS3-U3-41C6M, FLIR Integrated Imaging Solutions, Inc., Richmond, BC, Canada) is mounted on the top of the 3D Helmholtz coil system, in order to provide overviews for controlling the microswimmer, and to record videos for further observation, calculation, and analysis. The videos are recorded at a framerate of 50 frames/s.

### 3.2. Helical Swimmers with Different Hydrophobicity at Low Reynolds Numbers

The magnetically actuated miniature helical swimmers studied in the following experiments are made by Titanum. Each swimmer has 3.5 turns of helix with a pitch of 4mm, and a diameter of 3mm. The width of the helical tail is 1.2mm, and the thickness is 0.4mm. The cylindrical head is fabricated with a slot in the middle along its diameter, in which a cylindrical Rb-Fe-B permanent magnet is seated. The diameter of the magnet is 1mm, and the length is 3mm. The magnetization direction of the planted magnet is perpendicular to the body axis of the microswimmer, which enables it to be driven by a rotating magnetic field around its body axis.

**Figure 3:**
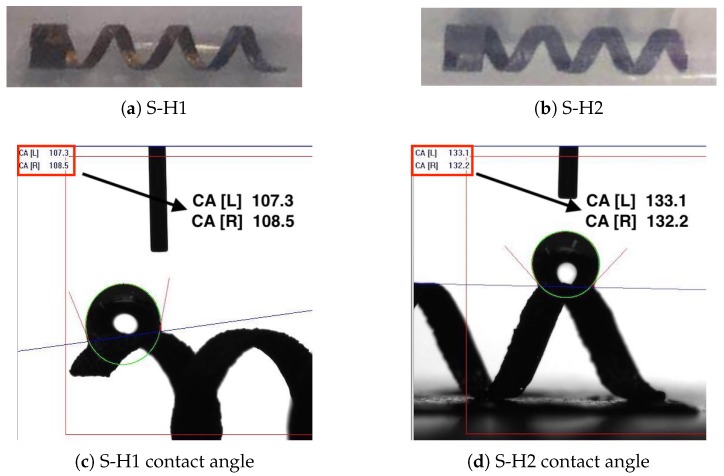
(**a**) S-H1: helical swimmer with less hydrophobic surface; (**b**) S-H2: helical swimmer with more hydrophobic surface. (**c**,**d**) The measured contact angles for S-H1 and S-H2.

The helical swimmers with different hydrophobicity were prepared through a potentiostatic anodization method with a two-electrode cell and followed by surface modification. Briefly, the titanium helical swimmer was washed with piranha solution for several minutes and dried in a drying oven before use. Then, titanium helical swimmer was used as the working electrode and the graphite rod was used as the counter electrode. Both electrodes were immersed in electrolyte solution contains 4% (*v/v*) HF and the mixture of Dimethyl sulfoxide (DMSO) and ethanal with a volume ratio of 1. The two electrodes were connected with a DC power supply with a voltage of 45 V for 1 5 h. After the reaction, the titanium helical swimmer with prepared surface TiO2 nanotube were washed with water. The dried sample was annealed at $450{\;}^{\circ }$C for 2 h to obtain anatase TiO2 nanotube. Finally, the titanium helical swimmer with high hydrophobicity (S-H2) was obtained from treating the sample in a 50 mL hexane solution of trimethoxymethylsilane (40μL) for 6 h, washed with hexane for three times, and dried in air. The titanium helical swimmer with less hydrophobicity (S-H1) was obtained from treating the sample in a 50 mL hexane solution of aminopropyltriethoxysilane (40μL) for 6 h, washed with hexane for three times, and dried in air. The standard deviation is calculated to be $4.9{}^{\circ }$ and $7.3{}^{\circ }$ for the less hydrophobic and hydrophobic swimmers, respectively. As is shown in Figure 3, the contact angles are measured respectively as $109.6{}^{\circ }$ for S-H1 and $133.4{}^{\circ }$ for S-H2. Thus, the helical swimmer S-H2 has a more hydrophobic surface.

A human spermatozoon moves with Re≈0.01. In order to make the swimmer in the similar Re numbers environment, the viscous solutions in this paper are selected and hydrophobicity influence on swimming performance is investigated. In the following experiments, the helical swimmers are designed to swim in glycerin solutions with glycerin percent weights of 85%, 92%, 96%, 98%, and 100%. Under $25{\;}^{\circ }C$, the density of 85% glycerin solution is measured to be $1.22\;g/{\mathrm{cm}}^{3}$, and the fluid viscosity is measured to be 78 mPa·s. The helical swimmers swim at a speed of approximately 0.5 mm/s to 2.5 mm/s, varying due to the difference in the rotation frequency of the applied magnetic field. According to Re=ρuL/μ, the Reynolds numbers in the following experiments are calculated to be approximately 0.01–0.1, thus the swimming behavior in 85% glycerin solution is in low Reynolds number regime. This also holds for 92%, 96%, 98%, and 100% glycerin solutions following similar calculations.

## 4. Experiments and Results

### 4.1. Measuring Swimming Velocity

Five sets of experiments were undertaken, with the variable among them being fluid viscosity. The glycerin percent weights were set to 85%, 92%, 96%, 98% and 100%, which resulted in an approximate arithmetic progression in fluid viscosity: 109, 310, 624, 939, 1410 (in centipoises, under temperature of 20), respectively. In each set of experiment, the swimmers S-H1 and S-H2 were separately put into the glycerin solution and actuated by a rotating magnetic field, which was generated by the 3D Helmholtz coils system. They are initially floating in the viscous liquid, and are actuated by a rotating magnetic field, which was generated by the 3D Helmholtz coils system. The propulsion velocity and step-out frequency are calculated before they sink down to the bottom. The rotation frequency of the magnetic field was initially set to 1 Hz, and was discretely increased by 1 Hz at a time. For each frequency value, the swimming behavior of the helical swimmers was recorded by the camera mounted on the top of the 3D Helmholtz coil system.

By fixing the recording frame rate to be 50 frames/s throughout the experiments, it is straightforward to establish a one-to-one correspondence between the time and the number of frames. The swimming velocity was measured by counting the number of pixels traveled along by the swimmer during a certain number of frames, followed by a unit conversion from pixels per frame into millimeters per second. In order to reduce experimental errors, measurements were conducted in five randomly selected disjoint time intervals, and the estimated velocity corresponding to a specific rotation frequency was obtained by averaging the five velocity values.

### 4.2. Impact of Hydrophobicity on Step-Out Frequency

**Figure 4:**
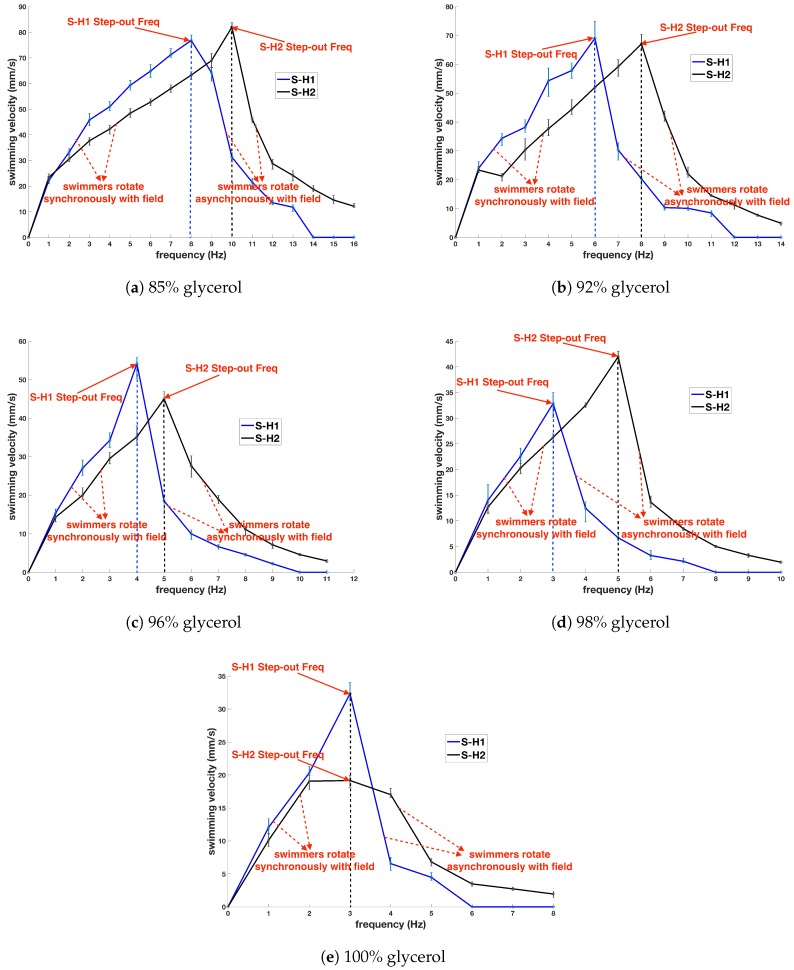
The propulsion velocity of S-H1 and S-H2 as a function of rotation frequency in different viscous liquids.

The propulsion velocity of S-H1 and S-H2 as a function of rotation frequency in different viscous liquids are presented in Figure 4. Each data point is associated with a pair of error bars, where the upper error bar indicates the difference between the estimated velocity value and the maximum value among the five sampling velocity values, and the lower error bar indicates the difference between the estimated velocity value and the minimum value among the five. For both S-H1 and S-H2, as the rotation frequency of the magnetic field increases, the swimming velocity first increases until reaching a peak, and then decreases towards zero. In the step-up section, the magnetic torque balances the fluidic drag torque, enabling the helical swimmer to rotate in sync with the magnetic field (i.e., the rotation frequency of the helical swimmer is identical to the rotation frequency of the magnetic field). Yet in the step-down section, the magnetic torque is insufficient to balance the fluidic drag torque, and therefore the helical swimmer rotates at a frequency lower than that of the magnetic field. Similar curve pattern is observed for glycerin solutions (liquid) with glycerin percent weights of 85%, 92%, 96% and 100%.

The step-out frequency is determined by identifying the frequency value corresponding to the peak of the curve, which is the last point where magnetic torque counterbalances fluidic drag torque. Under each of the five choices of glycerin percent weights, a larger step-out frequency is observed for S-H2, compared with that of S-H1. The difference in step-out frequency between the two helical swimmers verified that the hydrophobicity contributes positively to fluidic drag force (torque), so greater hydrophobicity results in smaller fluidic drag force (torque), and thus higher step-out frequency. Note that this result also matches the theoretical expectation obtained from Equation (14), which suggests a lower step-out frequency for the less hydrophobic helical swimmer.

### 4.3. Impact of Fluid Viscosity on Step-Out Frequency

As is discussed in Section 4.2, in a fluid with known viscosity, the step-out frequency of a helical swimmer can be obtained from the curve pattern of swimming velocity versus rotation frequency line graph. Note that the step-out frequency estimated in this way includes uncertainty, since there is a 1 Hz increment in applied rotation frequency between every two measurements of swimming velocity, yet the true value of step-out frequency is likely to fall between two integers.

**Figure 5:**
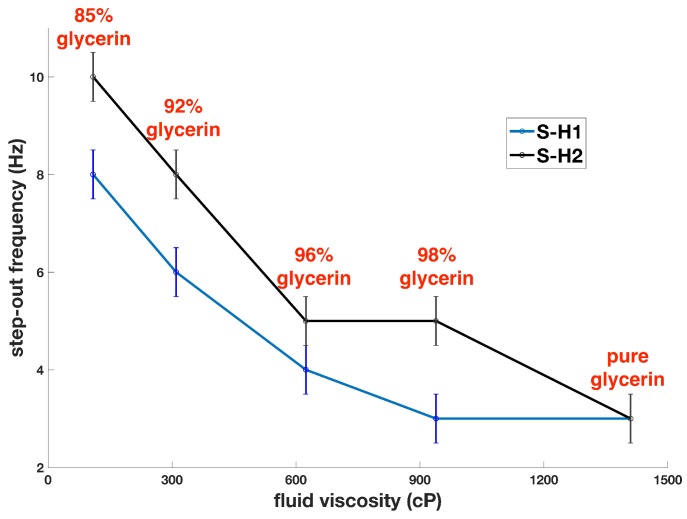
Step-out frequencies of S-H1 and S-H2 under increasing fluid viscosity.

To reveal the impact of fluid viscosity on step-out frequency, the step-out frequencies of the two helical swimmers with respect to fluid viscosity are depicted separately in a line graph, as is shown in Figure 5. With an increase in fluid viscosity, a general declining trend in step-out frequency is observed for both S-H1 and S-H2. Since fluid viscosity contributes positively to fluidic drag force (torque), greater viscosity results in greater fluidic drag force (torque), and thus lower step-out frequency. Given that fluid viscosity positively contributes to the shear stress $\tau_{w}$, the observed results also coincide well with Equation (14).

## 5. Conclusions

In this paper, we studied the swimming performance of miniature helical swimmers with different surface material hydrophobicity, and focused our attention on the difference in step-out frequency. Two millimeter-scaled swimmers were fabricated, with differently hydrophobic surface. Their swimming behavior was in low Reynolds number regime. Based on the balance between driving magnetic torque and resistive fluidic drag torque, as well as the fact that hydrophobicity negatively contributes to the shear stress, we raised a theoretical expectation that the less hydrophobic swimmer would exhibit a smaller step-out frequency. Experimental results obtained from glycerin solutions with different viscosity matched the theoretical analysis: in each set of experiment, the less hydrophobic swimmer showed a smaller step-out frequency compared with the more hydrophobic one. It has also been demonstrated that the influence of fluid viscosity on step-out frequency is negative: greater viscosity results in smaller step-out frequency. In future applications of helical microswimmers, modifying surface material hydrophobicity could be considered as a possible solution to improve the swimming performance.
